# Signaling Nodes Associated with Endoplasmic Reticulum Stress during NAFLD Progression

**DOI:** 10.3390/biom11020242

**Published:** 2021-02-08

**Authors:** Ja Hyun Koo, Chang Yeob Han

**Affiliations:** 1 Department of Physiology, College of Medicine, The Catholic University of Korea, 222 Banpo-daero, Seocho-gu, Seoul 06591, Korea; 2 School of Pharmacy and Institute of New Drug Development, Jeonbuk National University, 567 Baekje-daero, Deokjin-gu, Jeonju-si, Jeollabuk-do 54896, Korea

**Keywords:** ER stress, unfold protein response, nonalcoholic fatty liver disease, hepatocytes, hepatic stellate cells, Kupffer cells

## Abstract

Excess and sustained endoplasmic reticulum (ER) stress, paired with a failure of initial adaptive responses, acts as a critical trigger of nonalcoholic fatty liver disease (NAFLD) progression. Unfortunately, there is no drug currently approved for treatment, and the molecular basis of pathogenesis by ER stress remains poorly understood. Classical ER stress pathway molecules have distinct but inter-connected functions and complicated effects at each phase of the disease. Identification of the specific molecular signal mediators of the ER stress-mediated pathogenesis is, therefore, a crucial step in the development of new treatments. These signaling nodes may be specific to the cell type and/or the phase of disease progression. In this review, we highlight the recent advancements in knowledge concerning signaling nodes associated with ER stress and NAFLD progression in various types of liver cells.

## 1. Introduction

The endoplasmic reticulum (ER) is a central organelle that plays a critical role in a variety of fundamental biological processes, including protein synthesis and folding, lipid and cholesterol synthesis, xenobiotic metabolism, calcium storage and utilization, and providing a source of membrane for biogenesis of autophagosome and peroxisome [1,2]. Accumulation of misfolded or unfolded proteins in the ER lumen and disturbance of ER homeostasis overriding its capacity to handle this, a condition referred to as ER stress, induces the activation of unfolded protein response (UPR) in the cells [2,3,4]. UPR reduces the load of protein synthesis, but increases protein folding by chaperones with clearance capacity by promoting ER-associated degradation (ERAD) and autophagy [5,6,7]. Thus, while UPR is an adaptive physiological process that allows cells to maintain cellular homeostasis for survival, excess and sustained UPR can lead to pathological changes. 

ER stress is closely associated with various disease conditions, including metabolic disorders, neurodegenerative diseases, and cancers [8,9,10,11]. In particular, pandemic situation of obese populations and the importance of obesity as a key risk factor for metabolic diseases allow us to pay attention on the potential role of ER stress and UPR in metabolic dysfunction. The liver is one of the main metabolic organs that regulate energy homeostasis via its metabolic and secretory functions [12]. Hepatocytes are the most prevalent parenchymal cell-type in the liver (comprising 70–80% of total liver mass), which are enriched with ER due to liver functions such as protein synthesis and secretion. Indeed, numerous hepatotoxic challenges (e.g., hepatitis viruses, alcohol, drugs, and metabolic stress such as obesity and diabetes) can disturb ER homeostasis and ultimately lead to liver disease progression [13,14,15]. 

Nonalcoholic fatty liver disease (NAFLD) comprises a series of chronic liver disease ranging from simple steatosis to nonalcoholic steatohepatitis (NASH), which can progress to cirrhosis as more advanced stages and may ultimately lead to hepatocellular carcinoma (HCC) or liver failure [16,17]. Although NAFLD is recognized as a leading cause of liver-related morbidity and mortality, there are currently no drugs approved for its treatment [18,19]. Achieving a greater understanding of the cellular and molecular basis for the complex pathogenesis of NAFLD is a prerequisite for the discovery of novel therapeutic targets and strategies. In addition to the classical concept that lipid accumulation is the first hit and oxidative stress acts as the second hit for the disease progression, and it is now widely accepted that heterogeneous “multi-hits” are involved in the development of NAFLD [16,20,21]. Accumulating evidences enlightened ER stress as a major cause of multi-hits of NAFLD progression in multiple aspects such as hepatocyte injury, hepatic stellate cell (HSC), and immune cell activation [13,14,22]. Over the past decade, researchers have rigorously examined the effects of ER stress on liver pathophysiology and have collected strong evidence for a close relationship between ER stress and NAFLD. Unfortunately, the cellular and molecular mechanisms by which the body’s adaptive response failure to ER stress results in detrimental NAFLD progression are still largely unknown, particularly in aspects of downstream effectors and upstream regulators that may be involved. ER stress-mediated signaling pathways are distinct but complexly inter-connected with a dynamic regulation [23,24]. Moreover, the ER stress response also seems to play a critical role in various other non-parenchymal liver cells, supporting that precise understanding of UPR biology in a cell- and context-dependent manner is important. As general ER stress pathways have multifaceted effects on disease progression and resolution, targeting the specific effectors and/or regulators involved in NAFLD would be preferable. In this paper, we review recent advances in identifying the molecular basis for ER stress-mediated NAFLD and provide a summary of key signaling nodes in each cell type found in the liver.

## 2. Unfolded Protein Response Signaling: A Myriad of ER Stress Effectors

**Figure 1:**
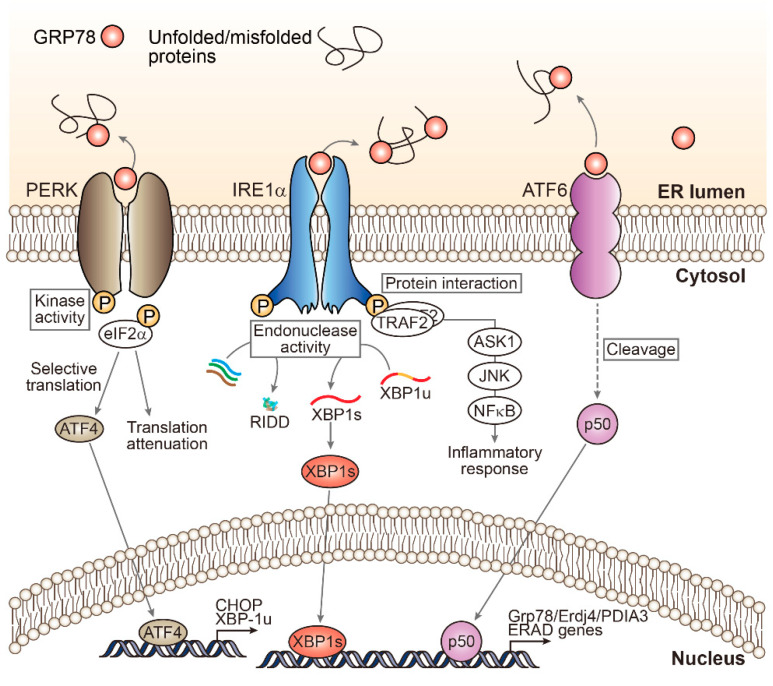
“Canonical” arms of endoplasmic reticulum (ER) stress signaling and unfolded protein response (UPR). The most upstream transmembrane kinases activated by ER stress. Although there is a myriad of signaling network triggered by ER stress, protein kinase RNA-like ER kinase (PERK), inositol-requiring enzyme 1 (IRE1α), and activating transcription factor (ATF6) ignite most of the downstream effector pathways. IRE1α degrades multiple mRNAs in a process called regulated IRE1α-dependent decay (RIDD), which results in reduced nascent protein load. Similarly, phosphorylation of eIF2α by PERK induces global attenuation of translation to slow down protein synthesis. Each of the three arms is capable of changing cellular transcriptome by distributing respective transcription factor(s) into the nucleus.

UPR is mediated by three canonical ER-resident stress sensors, protein kinase RNA-like ER kinase (PERK), inositol-requiring enzyme 1 (IRE1), and activating transcription factor (ATF6) [1]. When in their resting condition, these molecules bind to glucose-regulated protein 78 (GRP78) and keep it in an inactive state. However, all of these pathways can be activated after being dissociated from GRP78 in ER stress conditions, which affects diverse downstream events [2] (Figure 1).

PERK, a transmembrane protein with an N-terminal sensing domain and a cytosolic kinase domain, has eukaryotic translation initiation factor eIF2α as its major substrate [25]. eIF2α phosphorylation pauses general translation to reduce ER protein overload, but also increases protein translation elsewhere, including translation of ATF4 [26]. As a transcription factor, ATF4 induces gene expression of CCAAT-enhancer-binding protein (C/EBP) homologous protein (CHOP), a well-known downstream molecule of PERK-ATF4 [26,27]. CHOP and ATF4 redundantly and distinctly regulate the transcription of their target genes, which are involved in protein folding, redox homeostasis, autophagy, and apoptosis [27,28,29].

IRE1, the most conserved ER stress transducer, is unique in its possession of dual enzymatic activities as an endoribonuclease (RNase) and as a serine/threonine kinase [24]. As a consequence of the RNase activity of IRE1, the unconventional spliced form of X-box binding protein 1 (XBP1s) mRNA is produced [30]. This is a key transcription factor for the regulation of genes that encode for adaptive UPR, such as chaperones and ERAD [1]. In mice, the loss of either IRE1 or XBP1 causes embryonic lethality with severe defects in the liver [31,32]. Where ER stress is chronic, IRE1′s RNase activity also leads to the degradation of other mRNAs through regulated IRE1-dependent decay [33], which may contribute to pro-apoptotic signals by reducing the levels of several microRNAs [34]. IRE1 also activates the c-Jun N-terminal kinase (JNK) pathway downstream from tumor necrosis factor receptor (TNFR)-associated factor-2 (TRAF2)-apoptosis signaling kinase 1 (ASK1), which is associated with cell death and insulin resistance [13,30].

ATF6, an ER-resident transmembrane protein, is cleaved by proteases in the Golgi apparatus upon ER stress, and releases its cytosolic fragment that functions as a transcription factor with a basic leucine zipper motif [35]. The cleaved ATF6 then moves to the nucleus to regulate gene transcription for UPR [36]. While PERK and ATF6 are unessential for development of the liver, studies using global knockout mice suggested that they are required for rescue from pharmacological or dietary challenges [13].

While a number of researchers have focused on individual UPR pathways, these pathways are coordinately regulated, and thus have complex modes of action. From a pathological perspective, the numerous molecules involved in the UPR processes, as well as the associated upstream and downstream mediators, affect the presentation or progress of the disease at various stages. This highlights the importance of determining the specific molecular targets to understand and control the myriad of ER stress signaling on different diseases.

## 3. Clinical Association of ER Stress in NAFLD Patients

It has been observed that experimental ER stress inducers influence hepatocyte survival as well as intrinsic functions of hepatocytes such as de novo lipogenesis and glucose production. This realization recently prompted clinical researchers to perform liver biopsies on non-alcoholic and alcoholic fatty liver disease patients to examine the extent of ER stress.

In a study involving a small cohort of patients, mRNA and protein levels of GRP78 and CHOP were significantly elevated in livers of NASH patients [37]. Another study observed similar results in the livers of massively obese patients with simple steatosis or with NASH, which measured the ratio between CHOP and GRP78 mRNA expression as a readout of deleterious UPR [38]. Notably, in the studies hepatic ER stress was not induced in simple steatosis patient without apparent hepatocyte damage. These results suggest that ER stress plays a role in the phase transition from steatosis to steatohepatitis or fibrosis, but not in initial lipid accumulation per se.

While many of the signaling molecules in the UPR pathways are, by nature, Janus-faced, such that a change in ER stress marker expression may reflect either adaptive protection in the liver or disease progression, CHOP expression so far seems to be the most robust marker of detrimental direction. In a study using all serial stages of NAFLD, namely simple steatosis, NASH, fibrosis/cirrhosis, and HCC, hepatic CHOP expression was tightly correlated with progression of the disease [39]. Moreover, the contribution of CHOP in NAFLD progression has been proven by genetically deleted models using mice [39]. Prior to the recent reports, a previous study on NAFLD patients also has found increased PERK and IRE1α phosphorylation upon NASH progression. What remains unclear, however, is the process by which a signal propagates downstream as a result of ER stress, since changes in UPR molecules were not fully consistent to each other. To wit, eIF2α was phosphorylated, but ATF4 was unchanged, and JNK was activated but XBP1 splicing was unchanged [40]. Ultimately, the specific signaling molecule(s) that play a critical role in liver disease will need to be carefully evaluated for further therapeutic target development.

It is now clear that ER stress is clinically associated with NAFLD progression. In addition, considering that simple steatosis generally does not recruit ER stress and that lipid accumulation does not necessarily recruit hepatocyte death, there is mounting evidence from animal and human studies to suggest that ER stress plays a role in pushing the disease to advance from the fatty liver stage to inflammation or fibrosis.

## 4. Experimental Models Depicting ER Stress as the Key Trigger of NAFLD

The development of animal models that reflect the symptomatic and molecular patterns of human disease is necessary to facilitate the identification of therapeutic targets [41]. There have been a number of attempts to establish an ideal animal model that mimics each stage of human NAFLD. Widely used experimental models include dietary, chemical, or genetic interventions in mice [14,42]. Several models of NAFLD involved in increased ER stress, discussed as follow, are summarized in Table 1.

Models based on amino acid deficiency such as methionine-choline deficient (MCD) and choline-deficient, amino acid defined (CDAA) diets have long in use, and have the advantage of promptness and robustness. However, these models only partially reflect the stepwise progressive pattern of human NAFLD, in that early metabolic dysregulation observed in the disease is not accompanied in the experimental models even when fibrotic lesions begin to appear [41,42]. For this reason, recent research has tended to rely on obesogenic diets, including high-fat, high-carbohydrate (sucrose or fructose), or high-cholesterol diets. Obesogenic diets readily develop fatty liver and metabolic dysregulation (e.g., insulin resistance) and mirror the initial phases of human NAFLD. Unfortunately, these dietary modifications are often too mild, and it has proven difficult (or impossible) to induce severe symptoms such as fibrosis, cirrhosis, and HCC in models [41,42,43]. To circumvent this, several attempts have made to diversify dietary lipid composition to more closely reflect the so-called “western diet”. Accordingly, the American lifestyle-induced obesity syndrome (ALIOS) model was developed. It adds trans-fatty acids (45% fat) and cholesterol (2%) to the diet and induces steatosis and lobular inflammation with hepatocyte ballooning at 30 weeks of feeding [44]. While it imitates more physiological setting in terms of diet composition and symptomatic pattern, relatively slower onset is a limitation.

There also have been attempts to use chemical stimulation or genetic diversification to boost the relatively slow pathogenesis by dietary modifications. For example, streptozotocin administered neonatal mice (STAM) in addition to a high-fat diet (HFD), receive a single injection of streptozotocin 2 days after birth. STAM mice develop hepatic lipid accumulation and hepatocyte inflammation at a short time period (week 7) and promptly show fibrosis nodules (week 11–12) [45]. More recently, a western diet rich in fat, fructose, and cholesterol was combined with a weekly injection of low-dose CCl_4_ (0.2 μL/g) [46]. In this model, both the dose and frequency of the injection are less than half of what is typically used as a hepatotoxicant to induce hepatitis and fibrosis in mice. However, this combined model successfully induces stage 3 fibrosis at 12 weeks and development of cancer at 24 weeks. In addition, an inbred isogenic mouse strain produced by breeding 129S1/SvImJ strain with C57BL/6J strain has been developed to show age-dependent NASH and fibrosis, and termed the diet-induced animal model of nonalcoholic fatty liver disease (DIAMOND) model [47]. When the mice are fed with high-carbohydrate plus HFD, robust steatohepatitis with mild fibrosis appears at 16 week followed by bridging fibrosis at 52 week. Most importantly, large portion of DIAMOND mice eventually develop HCC. These newly developed models markedly resemble human NAFLD progression in their histological, metabolic, and transcriptional patterns, but studies are still needed to achieve consensus as to whether these new models are more suitable for translational research than their classically used counterparts.

MCD and obesogenic diets have also been widely relied on to study the link between ER stress and NAFLD. MCD diet feeding significantly increased eIF2α phosphorylation and CHOP expression in mouse liver, showing preferential activation of the PERK downstream signaling [48]. Progression to steatohepatitis was efficiently blocked by genetic ablation of CHOP [39]. Another study has found that high-fat high-sucrose diet feeding changes the ratio between phospholipid and free cholesterol in ER, which also correlated well with increased ER stress markers such as CHOP [49]. Increased CHOP expression was also implicated in an atherogenic diet model of NASH [50]. A separate study using a high-cholesterol diet showed morphological disruption of ER along with PERK phosphorylation [51]. In a similar context, a model *ob*/*ob* mice challenged with lipopolysaccharide (LPS) demonstrated that activation of both IRE1α and PERK and the resultant synergistic induction of CHOP coincided with NASH progression [38]. In sum, NAFLD experimental models reflect a strong concurrence between ER stress and disease progression. Alleviating ER stress by targeting downstream signaling molecule(s) can be effectively interrogated for NAFLD treatment using animal models.

Chemicals and genetic modifications that alleviate ER stress are being actively studied for liver disease treatment and prevention. Representative chemical chaperones that lessen ER burden against misfolded proteins include 4-phenyl butyric acid (4-PBA), a short-chain fatty acid, and taurine-conjugated ursodeoxycholic acid (TUDCA), a bile acid derivative. Administration of 4-PBA (1 g/kg/day; p.o.) or TUDCA (500 mg/kg/day; i.p.) alleviated hepatic steatosis development in *ob*/*ob* mice [52]. Liver injury and regeneration failure following partial hepatectomy and ischemia-reperfusion are also prevented by 4-PBS and TUDCA in rats [53]. Another mouse model using *ob*/*ob* mice injected with LPS has also proven the efficacy of TUDCA administration against NASH [38]. MUP-uPA mice that exhibit transient ER stress with high expression of urokinase plasminogen activator (uPA) specifically in the hepatocytes, were protected from HFD-induced steatosis and hepatocyte injury by TUDCA [54]. Most importantly, both 4-PBA and TUDCA have been approved by the Food and Drug Administration for the treatment of liver-related diseases (urea cycle disorder and primary biliary cirrhosis respectively), and following clinical trials for various disease are currently undergoing. Although the specific molecular mechanism how these chemicals act as chaperones is not fully understood, the studies have successfully proven that ER stress is one of the major potential therapeutic targets for NAFLD. Nonetheless, the molecular chaperones require high concentrations to lower ER stress, compromising suitability as a therapeutic agent.

## 5. Cell-Type Dependent Effects of ER Stress in Liver

**Figure 2:**
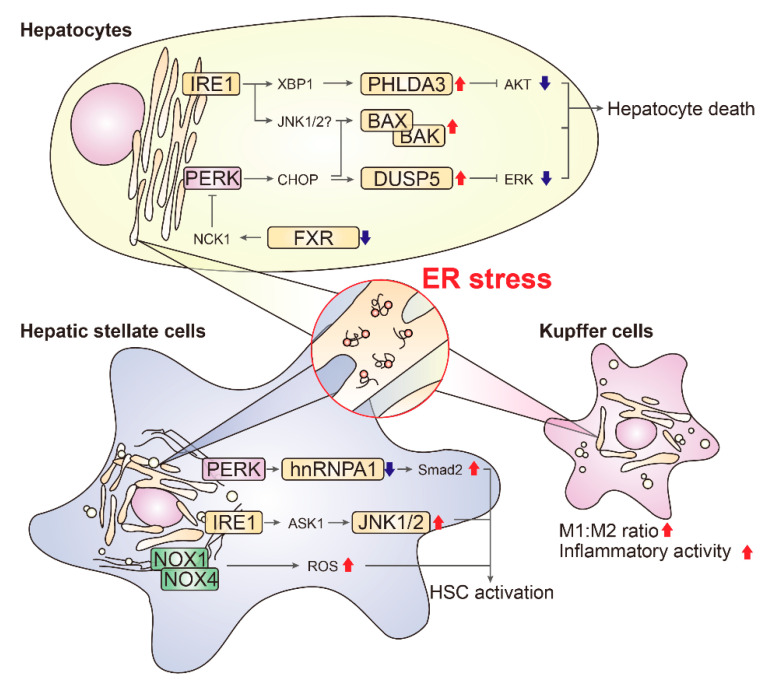
Cell-type specific molecular effectors of ER stress signaling in liver disease. ER stress-mediated signaling and the driver of pathogenesis varies across types of liver cells. Grey arrows indicate stimulatory (tipped) or inhibitory (blunted) interaction. Red upward and blue downward arrows indicate changes in key molecules or events that occur in cells that have experienced ER stress.

There is new evidence to suggest that the effects of ER stress vary by cell types. As a general rule, prolonged ER stress impairs parenchymal cells in each organ, leading to organ dysfunction or failure. However, some of the specialized cells that comprise non-parenchymal fraction of the tissues, namely hepatic stellate cells (HSCs) and macrophages in the liver, seem to be differentially regulated. These cells often secrete large amounts of protein and are sensitive to ER disturbance. Accordingly, ER stress has been regarded as an activating signal of non-parenchymal cells in the liver. In this section, we introduce some recently discovered ER stress signaling molecules that are involved in NAFLD progression, and which may be potential therapeutic targets (Figure 2).

### 5.1. Hepatocytes

#### 5.1.1. BAX/BAK

BCL-2-associated X (BAX) and BCL-2 homologous antagonist/killer (BAK) are pro-apoptotic members of the BCL-2 protein family [55]. Palmitic acid increased the expression of BH3-only proteins, BCL-2-like protein 11 (BIM) and p53 upregulated modulator of apoptosis (PUMA), through JNK and CHOP activation, leading BAX activation, and cell death in primary hepatocytes [56]. From this, we may discern that the mitochondrial apoptosis pathway, including BAX activation, can mediate fatty acid-induced lipotoxicity. However, in the livers of mice with double knockout of BAX and BAK, ER stress-induced tissue damages increased while IRE1 signaling decreased [57]. Similarly, a BAX chemical inhibitor (BI-1 or transmembrane BAX inhibitor motif containing 6 (TMBIM6)) acted as a negative regulator of IRE1 signaling [58,59]. While no inhibitor of BAX/BAK has yet been made available clinically, many attempts are being made [60,61,62], which may offer their use as a potential strategy for NAFLD treatment.

#### 5.1.2. ASK1

ASK1 is a serine/threonine kinase that links IRE1 to the JNK signaling pathway and is important for the regulation of cell survival and death [63,64]. Inhibiting ASK1 prohibited JNK activation and protected against hypoxia/reoxygenation injury in steatotic hepatocytes [65]. Based on the potential role of ASK1 in hepatic injury, inflammation, and fibrosis as determined by animal models, recently the safety and efficacy of selonsertib as a selective ASK1 inhibitor were evaluated in patients with NASH and stage 2 or 3 fibrosis. In this multicenter phase 2 trial, selonsertib improved the hepatic fibrosis of a considerable portion of the participants [66], raising the value of this target for potential NAFLD treatment. These results were consistent with multiple mouse model studies, in which ASK1 inhibition attenuated cell death and liver fibrosis in mouse models with liver injury caused by NACHT, LRR, and PYD domains-containing protein 3 (NLRP3) inflammasome activation [67]. A careful approach towards ASK1 inhibitors is needed; however, as it has also been reported that liver-specific ASK1 overexpression ameliorated hepatic steatosis and liver fibrosis through the induction of autophagy in mice [68]. Furthermore, the contribution of JNK signaling pathway as a downstream effector of ASK1 modulation in hepatocytes should be evaluated with caution, since multiple reports have pointed out hepatic stellate cells as the main place of deteriorative JNK signaling in the liver (also discussed in Section 5.2.2).

#### 5.1.3. PHLDA3

Pleckstrin homology-like domain, family A, member-3 (PHLDA3), is a unique protein with only a single PH domain, was initially proposed as a new target gene of p53 and a negative regulator of Akt signaling [69]. Accordingly, PHLDA3 has been considered as a tumor suppressor in primary lung cancers, pancreatic neuroendocrine tumors, and esophageal squamous cell carcinoma [69,70,71,72]. In a recent study, the novel role of PHLDA3 in liver pathophysiology was demonstrated. Hepatic expression levels of PHLDA3 were elevated in patients with liver diseases (e.g., HCV-infected hepatitis, HBV-associated acute liver failure, and liver fibrosis), which was validated in mice models with liver injury [73]. Interestingly, treating mice with an ER stress inducer increased PHLDA3 protein levels, especially in injured hepatocytes, and ER stress-augmented PHLDA3 gene transcription was mediated by the IRE1 and Xbp1s pathway in hepatocytes. PHLDA3 induction was account for Akt inhibition, which ultimately contributed to hepatocyte death and liver injury. The fact that PHLDA3 can be found in urine [74] raises its value as a potential diagnostic and/or prognostic biomarker, and suggests that it has potential as a new therapeutic target for liver diseases that are exacerbated by ER stress. It is noteworthy that, in extrahepatic conditions, PHLDA3 deficiency improved cell viability during early islets transplantation [75], but its silencing also potentiated cytokine-induced apoptosis of beta-cells [76]. Additional investigation may reveal further details concerning the role of PHLDA3 in various liver diseases associated with ER stress.

#### 5.1.4. DUSP5

Phosphorylation is one of the key post-translational modifications (PTMs) responsible for the regulation of signal transductions including UPR. The phosphorylation status of the proteins is regulated by balance between kinases and phosphatases; thus, deregulation of the actions of phosphatases as a fine-tuner can lead to pathological conditions. Dual-specificity phosphatases (DUSPs) mediate dephosphorylation of their targets at sites of serine/threonine and tyrosine residue [77]. The substrates of DUSPs include mitogen-activated protein kinases, which are involved in the regulation of oncogenic transformation and immune responses [77,78,79]. Recently, DUSP5 was proposed as a potential target for ER stress-mediated hepatocyte injury [80]. DUSP5 expression was upregulated in patients and mice with liver fibrosis, and induced by ER stress via the PERK–CHOP pathway in hepatocytes. The DUSP5 induction reduced ERK activity and facilitated hepatocyte death. In vivo validation of these effects is needed, but these initial results are certainly worth following up.

#### 5.1.5. FXR

Farnesoid X receptor (FXR, NR1H4) is a nuclear receptor with bile acids as an endogenous ligand, which regulates the metabolism of bile acids, lipid, and glucose [81,82]. In a study of aged mice, ER stress repressed hepatic FXR expression levels via the inhibition of hepatocyte nuclear factor 1α [83]. Recently, FXR was identified as a negative regulator for ER stress-mediated NLRP3 inflammasome activation [84]. NLRP3 inflammasome activation by ER stress contributed to hepatocyte death, inflammation, and ultimately liver fibrosis [38,85]. The hepatic expression of FXR was reciprocally changed to NLRP3 components and interleuckin-1β as a key product of the inflammasome activation in patients with liver disease including NAFLD [84]. In mice, FXR deficiency exacerbated NLRP3 inflammasome activation and exaggerated subsequent liver damage from ER stress challenges, while treatment with an FXR agonist had a protective effect [84]. Mechanistically, FXR suppresses microRNA-186, an inhibitor of the non-catalytic region of tyrosine kinase adaptor protein 1 (NCK1) translation, leading to the inhibition of the PERK pathway [84]. It supports the role of FXR as an upstream regulator of the ER stress pathway. As a number of studies have reported the beneficial effects of FXR on liver and metabolic diseases, it has become an attractive target for potential drug development. Obeticholic acid, a semi-synthetic FXR agonist, is one of many promising candidates for the treatment of NAFLD, although there are some concerns over the cardiovascular effects of the drug [82,86].

### 5.2. Hepatic Stellate Cells

The activation of HSCs into proliferative, fibrogenic myofibroblasts is well-established as the central driver of hepatic fibrosis in both experimental models and humans [87,88]. ER stress increases expression of fibrogenic genes in HSCs and stimulates myofibroblastic conversion [89,90]. For example, XBP1 induces expression of collagen type I in HSCs downstream of IRE1 arm, which is inhibited by knockdown of ATG7 [91]. In contrast, reducing the ER burden placed on misfolded proteins by HSC-specific overexpression of GRP78 under the α-SMA promoter effectively reduced CCl_4_-mediated fibrosis [89]. Similarly, partially inhibiting UPR signaling by dominant-negative mutant IRE1α in HSCs also prevented their activation [90]. During the initial phase of myofibroblastic conversion, acute ER stress induction was briefly observed [92], but the exact mechanism remains unclear.

#### 5.2.1. SMAD2

The PERK arm of the ER stress downstream signaling pathway is a major contributor to HSC activation. Specifically, ER stress in HSCs promotes liver fibrosis by inducing fibrogenic gene expression through PERK-mediated induction of SMAD2 [89], a powerful driver of fibrogenesis downstream of TGFβ. PERK directly phosphorylates and destabilizes RNA-binding protein HNRNPA1. HNRNPA1 binds to unprocessed miRNA, preferentially to pri-miR-18A, to promote its splicing and maturation. The PERK-induced decrease in HNRNPA1 subsequently decreases miR-18A levels, resulting in derepression of its mRNA counterpart, SMAD2. Importantly, lentiviral delivery of HNRNPA1 in HSCs alleviates fibrosis progression in a murine model using CCl_4_ [89]. While this result is promising, additional efforts are needed to identify and establish a druggable target capable of controlling this pathway.

#### 5.2.2. JNK1/2

JNK1/2 are activated downstream of PERK and IRE1α, and have long been implicated in the mediation of ER stress-related hepatocyte death and steatohepatitis [93]. The kinase activity of IRE1α contributes to JNK activation through TRAF2-mediated ASK1 recruitment, and PERK is capable of activating JNK [94,95,96]. JNK1/2, however, mainly acts on HSC, not on hepatocytes, at least during the fibrosis stage [97]. In a mouse study, chemical inhibition of JNK1/2 did not reduce hepatocyte death, but did prevent fibrosis. JNK1 knockout mice were more resistant to fibrosis development while this effect was not observed in JNK2 knockout, identifying JNK1 as a determinant HSC-activating kinase. Moreover, genetic ablation of JNK1 in mice rescued from fibrosis development. Notably, the dominant contribution of JNK1 in HSCs was further confirmed by tests of hepatocyte-specific JNK1 deletion in mice [98].

#### 5.2.3. NOX1/NOX4

A manageable level of reactive oxygen species (ROS) are produced during physiological ER activity. Stressed ER amplifies ROS production through transmembrane NADPH oxidases (NOXs). In addition, another contributor of ROS generation under ER stress condition is ERO1 as an ER oxidase. CHOP induces the expression of ERO1 and thereby increases abnormal oxidized proteins [99]. In rats, chronic arsenic (NaAsO_2_) ingestion paired with ER stress induced oxidative stress and HSC activation [100]. In HSCs, IRE1α-mediated activation of NOX1/NOX4 and excessive ROS production induced fibrogenic activation. Conversely, the inhibition or deficiency of either NOX1 or NOX4 effectively reduced inflammation and fibrosis in mice [101]. At present, an orally available NOX1/NOX4 inhibitor (GKT137831) is undergoing clinical trials for pulmonary fibrosis [102,103]. Therefore, NOX inhibition may be one of the most feasible strategies.

### 5.3. Kupffer Cells

Kupffer cells (KCs), inherent hepatic immune cells, play pro- and anti-inflammatory role in the liver, and contribute to the progression of steatohepatitis. KCs are divided into interchangeable subtypes; pro-inflammatory (M1) and anti-inflammatory (M2). ER stress in KCs was activated during NAFLD [14,104]. Moreover, it has been suggested that simple steatosis as well as palmitate treatment in vitro recruits ASK1 activation downstream of IRE1α only in KCs, but not in hepatocytes [65]. Specifically, the populations of M1 KCs as well as inflammatory cytokines such as interleukin (IL)-6, IL-1β and tumor necrosis factor (TNF)-α were increased by ER stress [105]. Conversely, inhibiting UPR signaling by treating chemical chaperone 4-PBA or inhibiting IRE1α by genetic knockdown or chemical inhibition increased M2 population and decreased M1 population. While these results are interesting and justify further investigation, it must be noted that the signaling molecules(s) downstream of ER stress that are responsible for specifically regulating inflammatory activity of KCs during NAFLD are not well understood.

## 6. Conclusions

ER stress is a significant trigger of NAFLD progression, which follows hepatic lipid accumulation. Therapies designed to inhibit ER stress by interfering with its associated signaling, such as broad-spectrum ER stress-reducing agents (4-PBA or TUDCA) and targeting canonical UPR branches (PERK or IRE inhibitors), have been tested as potential treatments for liver diseases [106,107]. Other agents that directly interfere with UPR signaling may provide potential therapeutic benefits, though most remain to be evaluated in the context of NAFLD. What is clear is that given the protective nature of UPR signaling, the redundant inhibition of ER stress in non-diseased cells or tissues may compromise the benefits. Ultimately, achieving the targeted regulation of the key molecule(s) that drive pathogenesis is a more desirable solution. While many possible approaches to reaching this goal have been briefly covered in this review, careful translational validation is needed to extrapolate the data to clinical trials. Identification of the novel signaling molecules that link ER stress and the disease pathogenesis is of equal importance. Finally, further in-depth characterization of the cell-type specific or context-specific regulatory mechanisms of UPR and key molecules will provide information valuable to the efforts of researchers to develop new means of controlling and treating diseases.

## Figures and Tables

**Table 1:** Experimental nonalcoholic fatty liver disease (NAFLD) models with ER stress activation.

Model	Altered ER Stress Marker	Human NAFLD Reflection	Effect of UPR Intervention
MCD diet	p-eIF2α↑ CHOP↑ GRP78↑	NASH and fibrosis, without steatosis	TUDCA administration (500 mg/kg/day) prevented liver injury CHOP KO reduced NASH progression
High-fat diet (20 weeks)	p-PERK↑ CHOP↑	Steatosis, NASH, and mild fibrosis	
High-fat, high-sucrose diet	XBP1s↑ CHOP↑ ER free cholesterol/phospholipid ratio↑	Steatosis and mild NASH and fibrosis	
Atherogenic diet	CHOP↑	Steatosis, NASH, and fibrosis	
High-cholesterol diet	ER morphological disruption p-PERK↑	Steatosis, NASH, and mild fibrosis	
ALIOS model	CHOP↑	Steatosis, NASH, and fibrosis	
DIAMOND mice	CHOP↑	Steatosis, NASH, and fibrosis	
*ob*/*ob* (in combination with LPS)	GRP78↑ XBP1s↑ p-eIF2α↑	Steatosis, NASH, and fibrosis	TUDCA administration (500 mg/kg/day) prevented steatosis, hepatocyte death and inflammation
MUP-uPA (in combination with HFD)	GRP78↑ p-eIF2α↑ CHOP↑ p-IRE1α↑ p-JNK1/2	Steatosis, NASH, and fibrosis	TUDCA administration (250 mg/kg/day) reduced steatosis and hepatocyte death
Chronic CCl_4_	GRP78↑ p-PERK↑ CHOP↑	NASH and fibrosis, without steatosis	Targeted lentiviral delivery of GRP78 in hepatic stellate cells reduces fibrosis
